# Edible Bird’s Nest: Recent Updates and Industry Insights Based On Laboratory Findings

**DOI:** 10.3389/fphar.2021.746656

**Published:** 2021-10-01

**Authors:** Kian Chung Chok, Ming Guan Ng, Khuen Yen Ng, Rhun Yian Koh, Yee Lian Tiong, Soi Moi Chye

**Affiliations:** ^1^ School of Health Science, International Medical University, Kuala Lumpur, Malaysia; ^2^ School of Pharmacy, Monash University Malaysia, Selangor, Malaysia; ^3^ Division of Biomedical Science and Biotechnology, School of Health Science, International Medical University, Kuala Lumpur, Malaysia; ^4^ School of Postgraduate, International Medical University, Kuala Lumpur, Malaysia

**Keywords:** edible bird’s nest, industry insights, laboratory findings, saliva of swiftlets, bioactive compounds, nutritional contents

## Abstract

Edible bird’s nest (EBN) is a traditional Chinese delicacy made of the saliva of swiftlets found in Southeast Asia. With increasing demands for EBN, quality control of EBN products is important for safe consumption. The processing steps are particularly important for efficient extraction of bioactive compounds. Geographical location, collection place, and harvesting season contribute to differences in nutritional contents in EBN. Concerns regarding presence of adulterant, chemical, and microbial contaminants in EBN as well as authentication and chemical composition measuring methods are discussed in this review. Recent discoveries of beneficial health functions of EBN in antimicrobial and antiviral actions, immunomodulation, cancer prevention and treatment, tissue regeneration, cardiometabolic maintenance, antioxidant action and neuroprotection are also reviewed. Our review provides an update on the recent research on EBN.

## Introduction

Consumption of edible bird’s nest could be traced back to the 7^th^ century from China, in which EBN was classified as a high-grade health food, tonic, medicine, and a symbol of wealth (Lau and Melville, 1994). Traditional Chinese medicine states that EBN confers health benefits such as moisturizing the lung, resolving phlegm, alleviating cough, and recuperating from diseases (Lim and Earl of Cranbrook, 2002; Hobbs, 2004). EBN is usually referred to the nests of *Aerodramus fuciphagus* (*A. fuciphagus*) (white-nest swiftlet) and *Aerodramus maximus* (*A. maximum*) (black-nest swiftlet) harvested for consumption in Southeast Asia (Hao et al., 2015). The growth and reproduction of swiftlets require appropriate environmental conditions such as humidity of about 80–90%, temperature between 26 and 35°C and sufficient food sources. Hence, EBN-producing swiftlets are mainly found in Southeast Asian countries such as Malaysia, Thailand, Indonesia, Myanmar, Vietnam, The Philippines, Cambodia, and the southern region of China. However, the quality, composition, and nutritional values in EBN could be different based on the swiftlet species, geographical as well as environmental conditions (Dai et al., 2020). Increasing demands for EBN has produced economic value from US$1000.00 to US$10,000.00 per kilogram depending on its grade, shape, species, and origin. Indonesia is the largest bird’s nest producer in Southeast Asia, exporting around 2,000 tons/year, followed by Malaysia at 600 tons/year, and Thailand at 400 tons/year (Panyaarvudh, 2018). China is the largest EBN importer every year. From January to June 2021, China imported approximately 128.3 tons, 42.3 tons, and 0.1 tons from Indonesia, Malaysia, and Thailand respectively, in the form of ready-to-drink beverages and processed EBN (Development Report of Imported Bird’s Nest in 2021, 2021). Indonesia exported about US$998 million worth of EBN in 2015 and the value surged up to US$3.64 billion in 2019 (Badan Pusat Statistika, 2020). EBN production in Myanmar is comparatively lower as the industry has just begun a decade ago by building the first EBN house in Bokpyin, southern Myanmar (Big business in bird’s nest in Myanmar seaside town, 2017). In China, there are 3 well-known places with swiftlets inhabitant: Huaiji County, Guangdong province, which produces 150 kg/year; EBN collection is prohibited on Dazhou Island, Hainan because swiftlets are listed as endangered species; EBN produced from Jianshui County, Yunnan is only for the consumption by local residents (Geographical distribution of swiftlet inhabitant, 2020). Due to increasing demands for EBN, quality control should be in place to ensure safe consumption of EBN. Studies that investigated the nutritional value, adulterant content, nitrite (NO_2_^−^), nitrate (NO_3_^−^), and microbial contamination in EBN have increased in recent decade (Lee et al., 2021). Our review will discuss on EBN extraction methods, nutritional values, health benefits, and the risk of EBN consumption by including the latest findings about EBN. There is an existing review about EBN which includes publications up to 2019 indexed in Web of Science (Lee et al., 2021). Our review aims to provide an update by including those publications which were not discussed in previous reviews and discuss on additional topics including industrial EBN processing based on laboratory findings. This is a narrative review which includes latest EBN publications up to April 2021. Published data were retrieved from PubMed, Semantic Scholar, Meta, X-MOL, and Google Scholar, using search string: edible bird’s nest.

## Chemical Compositions of Edible Bird’s Nest

The quality of EBN is based on various factors such as species of swiftlets, habitat (man-made house or natural cave), harvesting season (rainy season or drought season), and geographical location (Malaysia, Thailand, Indonesia, Myanmar, and China). Quality of EBN is usually screened and maintained at the stage of industrial preparation. Despite the challenges and limitations, numerous methods were applied to ensure authenticity, nutritional values, and safety for EBN consumption, ranging from empirical measures to molecular biology-based techniques.

### Factors Affecting Chemical Compositions of Edible Bird’s Nest

Nutritional and authenticity studies show that EBN acquired from different sources, such as swiftlet premises, natural caves, and retail stores contain significantly varied chemical compositions. The *A. fuciphagus* EBN from swiftlet premise has higher antioxidant activities and sialic acid content whereas the *A. maximus* EBN from the cave has more mineral composition of calcium and magnesium. Total amino acids in *A. fuciphagus* EBN was found to be 23% higher than *A. maximus* EBN (Quek et al., 2018a). A simple gel electrophoretic method could be employed to differentiate house and cave EBN by generating a fingerprint profile of EBN. The cave EBN produces 10 bands with 2 strong bands at 30 and 35 kDa, house EBN produces 9 bands with 2 strong bands at 120 and 140 kDa (Hun et al., 2016). These results show different protein contents in EBN from different origins and hence there is a need to further investigate this difference in protein composition. Cave EBN was also reported to have 3 times more calcium content than house EBN. It is believed that the moist limestone cave wall provides abundance Ca^2+^ to seep into the cave EBN whereas the house EBN is built on timber strips that have no Ca^2+^ to leach out. (Shim and Lee, 2020). In a field study, cave EBN collected from small islands in Indonesia and Malaysia contain higher NO_3_^−^ and NO_2_^−^ levels when compared to house EBN. It is probably due to the symbiotic relationship between plants and nitrogen-fixing bacteria providing space (i.e., root nodules) for the growth of nitrogen-fixing bacteria in exchange for NO_3_^−^ as nutrients. It is speculated that nitrogen-fixing bacteria reaches the mouth of swiftlets when the swiftlets prey on insects or directly from plants or soil (Schultze and Kondorosi, 1998; Chan et al., 2013). The risk of NO_3_^−^ and NO_2_^−^ contamination will be discussed later in this review.

Nutritional compositions in EBN are known to be affected by the harvesting season. Swiftlets feed on insects from *Hymenoptera* (winged ants, fig wasps and bees), *Coleoptera* (small beetles), *Homoptera* (leafhoppers) and *Ephemenoptera* (mayflies) species. The food sources for swiftlets are more abundant during the rainy season. EBN protein content ranged from 60.3 to 63.6 g/100 g for samples from North Peninsular Malaysia and 57.9–61.2 g/100 g from the East Coast Peninsular Malaysia with the highest protein level found in samples collected during December to March. For EBN samples obtained from South Peninsular Malaysia, protein content ranged from 61.8 to 65.2 g/100 g and samples harvested from August to November had the highest protein content. However, the mineral contents found in EBN samples from all different zones are not affected by the season (Norhayati et al., 2010).

Farmed EBN along the seashores of Thailand were reported to be rich in sulphur-containing amino acids, calcium, and magnesium (Saengkrajang et al., 2013). The use of Gas Chromatography-Mass Spectrometry (GC-MS) and Liquid chromatography-Mass Spectrometry (LC-MS) together with orthogonal projection to latent square discriminant analysis (OPLS-DA) could identify the origins of EBNs accurately based on the fatty acid content in the Malaysian, Indonesian, and Thailand EBNs. Generally, the levels of fatty acids and fatty acid amides are highest in Malaysian EBNs followed by Indonesian and then Thai EBNs. Moreover, the Malaysian and Indonesian EBNs could be differentiated with distinct fatty acids profiles in which the fatty acids of Malaysian and Indonesian EBNs are separated along the PC1 axis in the loading plot. (Chua et al., 2014). Thailand has a huge land mass cultivating rice whereas Malaysia and Indonesia have large palm oil plantations (Seeboonruang, 2012; Meijaard et al., 2020). Swiftlets usually feed on insects nearby their habitats, the swiftlets in Thailand feed on insects with a rice-based diet, resulting in low fatty acids profile in Thai EBN. In contrast, the swiftlets in Malaysia and Indonesia feed on insects with a diet rich in palm oil, resulting in high fatty acids content in their EBNs (Lourie and Tompkins, 2000; Zhou et al., 2003; Tres et al., 2013). In the same study by Chua et al., the cave EBN contains higher level of ergosterol, a component of fungi and heptadecasphinganine, antifungal agent when compared to house EBN indicating higher amount of fungi in cave EBN and production of heptadecasphinganine by the swiftlets (Chua et al., 2014).

EBN is seen to be rich in protein, essential amino acids, essential trace elements, and essential sugars for human biological functions. Pure EBN collected from swiftlet premises in Batu Pahat, Malaysia contains carbohydrate (46.47%) (w/w), protein (35.80%) and a very minute fat content (1.30%). EBN contains relatively high amount of sodium (6017 mg/kg), magnesium (344 mg/kg), potassium (138 mg/kg) and calcium (68 mg/kg). Other minerals such as phosphorus, iron (Fe), chromium, and selenium are 0.037, 4.52, 0.30 and 0.14 mg/kg respectively and trace amounts of heavy metals arsenic (As) (0.0237 mg/kg), lead (Pb) (0.0203 mg/kg), copper (Cu) (0.6783 mg/kg) and zinc (1.2542 mg/kg) were found in the EBN. Malaysia Food Act 1983 (Act 281), Part VII (Incidental Constituent), Regulation 38 consider them as heavy metals. However, these heavy metals were within the infant formula specification limit as set by the Act. No mercury (Hg) and cadmium (Cd) were detected in the EBN. Fibre would only be detected in EBN if adulterant containing vegetative matter was added into EBN (Lee et al., 2015). Proximate nutritional compositions were compared between *A. fuciphagus* EBN from Pahang and Terengganu, Malaysia (Halimi et al., 2014).

Systematic analysis of nutritional composition of EBN collected from different regions in Indonesia was recently conducted. EBN collected from West Sumatra, South Sumatra, West Java, West Kalimantan, Central Sulawesi, and Southeast Sulawesi were analysed. The proximate nutritional compositions are protein (53.09–56.25%), carbohydrate (19.57–23.04%), moisture (17.08–21.50%), ash (5.44–6.25%), and fat (0.07–0.57%). EBN collected from different locations in Indonesia contain 18 types of amino acids, including 10 essential amino acids and 8 non-essential amino acids, ranging between 16.15 and 20.88%. NO_2_^−^ contents were found to be ranging from 3.11–18.28 ppm (average 8.40 ppm) (Elfita et al., 2020).

EBN from Myanmar was found to contain high protein (53.5–59.3%), sodium (0.17%), potassium (237.1 ppm), calcium (0.71%), magnesium (361.3 ppm), and zinc (12.3 ppm). The NO_2_^−^ and NO_3_^−^ levels in EBN were estimated by measuring NO_2_^−^ and NO_3_^−^ contents in filtrate water after soaking with EBN for 4 h. The NO_2_^−^ and NO_3_^−^ levels in filtrate water are 0.27 and 4.0 ppm respectively (Phyu Win et al., 2020).

### Authentication of Edible Bird’s Nest

In the past, EBN was considered 'Caviar of the East' and was expensive (from US$1000.00 to US$10,000.00 per kilogram).There have been incidents of adulterants added into EBN during the processing to increase the final weight of EBN product. The addition of adulterants possesses risks to the health of EBN consumers as well as economical loss to the consumers when faulty goods were purchased (EBN products containing adulterants). Common adulterants such as tremella fungus (*Tremella fuciformisis*), jelly, fish swimming bladder, egg white, pork skin, karaya gum (*Sterculia urens*), and red seaweed could cause unwanted health effects and allergy if consumed unnoticeably (Thomas et al., 2019; Yeo et al., 2021). The relevant government departments and companies are responsible to authenticate and prevent consumers from consuming EBN products containing health-threatening adulterants.

Gel electrophoretic protein fingerprint profiling could be done to differentiate EBN from white fungus, jelly, fish swimming bladder and egg white. Cave EBN (30 and 35 kDa), house EBN (120 and 140 kDa), white fungus (22, 35, and 75 kDa), egg white (35 and 75 kDa), fish swimming bladder (streaking bands), and jelly (no band). The amino acid fingerprint profile of cave EBN, house EBN, white fungus, egg white, fish swimming bladder, and jelly are all distinct from each other and could be identified by HPLC (Hun et al., 2016). Proximate nutritional analysis shows that addition of adulterants, such as karaya gum, red seaweed, and tremella fungus, could account for 2–10% of the final weight of EBN and reduce the crude protein content of EBN up to 6.2% (Marcone, 2005). Authentic EBN contains more than 3 mg epidermal growth factor (EGF) in 1 g of protein as detected using a simple immunoblotting assay (Yang et al., 2014). Guo et al. established a TaqMan-based real-time polymerase chain reaction (PCR) assay to specifically detect EBN components and 4 common adulterants: white fungus, agar, pork skin and egg white. Five sets of primers and probes were designed for these five components. The assays were specific and reproducible even after the samples have undergone vigorous, tedious, and complicated processing. The relative detection sensitivities were 0.5% EBN, 0.001% white fungus, 0.5% agar, 0.001% fried pork skin and 1% egg white (Guo et al., 2014).

Metabolite profiling refers to holistic analysis of the molecular information of food which has recently gained attention in the food industry (Xu et al., 2006). The use of mass spectrometry coupled with chromatography resulted in improved data quality and sensitivity (Chua et al., 2014). The mass spectrometry method was further improved using GC-MS and LC-MS together with the chemometrics model, orthogonal projection to latent square discriminant analysis (OPLS-DA). These methods could classify the EBNs based on production site, colours, and countries. OPLS-DA has the advantage of being able to predict the identity of unknown samples. GC-MS coupled with OPLS-DA was discovered to be a better EBN authentication method for quality control of EBN compared to LC-MS, of which detection of the metabolite could be affected due to EBN processing (Chua et al., 2014). GC-MS was used to detect the oligosaccharides content in EBN, especially the N-Acetylneuraminic acid (NANA). By using environmental scanning electron microscopy, the micrographs of authentic raw EBN samples display the crater-like structure of irregularly shaped three-dimensional networks, measuring from less than one, up to several microns. However, the fake EBN samples made from jelly fungus, agar, and pork skin display granule- or cudgel-like structures rather than network structures. In another study, well-established gas chromatography with flame-ionisation detection (GC-FID) was employed to analyse and quantify amino acids in EBN whereas optimised liquid chromatography with tandem mass spectrometry (LC-MS-MS) was applied for monosaccharide analysis. The data generated were combined with Hotelling T2 range plot to identify EBN and non-EBN. Hotelling T2 range plot is a chemometrics plot that reduces the amino acids and monosaccharides into a T2 value. This plot simplifies data analysis process and, more importantly, combines multiple variables to create a unique fingerprint for the sample (Chua et al., 2015). Its application is often found in the quality control of products (Eppe and De Pauw, 2009). This method has since then discovered EBN to contain a group of glycoproteins that are not affected by the EBN’s colouration, country of origin, and/or the processing method of the food item (Chua et al., 2015).

Samples damaging during authentication of EBN is the major concern in the authentication process. Contamination or loss of chemical composition could result in false reporting if samples were not handled with care. Thermogravitiy (TG) and differential thermogravity (DTG) techniques require 5–10 mg of EBN samples for rapid authentication of EBN by comparing the TG and DTG curves produced from EBN and adulterated EBN. EBN was heated in the crucible from 25°C to 1000°C and 800°C for unadulterated and adulterated samples to generate the TG and DTG curves. TG is presented in the curve of mass percentage versus temperature whereas the first derivative DTG curve gave the rate of change of mass loss percentage versus temperature. Hence, adulterated EBN (e.g. glucose, sucrose, hydrolysed marine collagen, and monosodium glutamate) has distinctive TG and DTG curves which could be identified when compared to TG and DTG curves of pure EBN (Shim et al., 2017). Shi et al. (2017) designed a rapid and non-destructive method to characterise the distribution map of carbohydrates, proteins, and sialic acid in EBN using hyper-spectral imaging. The chemometrics and spectral signals of EBN were used to build calibration models which then were tested by extracting and predicting spectra of each pixel in EBN hyperspectral images. The distribution map of carbohydrates, proteins, and sialic acid in EBN were characterised with unevenly distributed carbohydrate and protein contents based on the swiftlets diet consumption: Insects and small fish consumption increased protein content whereas seaweed consumption increased carbohydrates content. However, sialic acid is evenly distributed in the entire EBN (Shi et al., 2017). Future studies on these methods to identify adulterated EBN and their potential application in industry EBN preparation is promising to be ventured.

Fourier Transform Infrared (FTIR) spectrum of the raw unprocessed EBN was identical to that of the spectra of processed EBN samples. The spectra of adulterated EBN samples were different from that of processed EBN. Fingerprint region of the spectra of pure edible bird and adulterated edible bird nest samples were different at <1700 cm^−1^. The NH group was absent in all adulterants except in pork skin and egg white. Interestingly, only pork skin has ester C=O stretch bond and C=CH group which were not present in EBN. Hence, this technique could be used for testing the authenticity of the EBN samples as well as detection of porcine products (Hamzah et al., 2013). Apart from that, indirect enzyme-linked immunosorbent assays (ELISAs) was developed to detect porcine gelatine adulteration using anti-peptide polyclonal antibodies. Three indirect ELISAs were developed for porcine species-specific amino acid sequences of the collagen (I) α2 chain (PAB1 and PAB2) and the collagen (I) α1 chain (PAB3), which had limits of detection of 0.12, 0.10 and 0.11 µg/g respectively (Tukiran et al., 2015).

Environmental factors, such as locations, harvesting places, and harvesting seasons could affect the quality of EBN and a standardised guideline is required for quality control of EBN products. Differences in nutritional, adulterants, and contaminants detected could be due to different EBN handling, beginning from collection, processing, delivery, and storage. The EBN industry in Malaysia and Indonesia suffered a severe blow in 2011 when China banned exportation of EBNs due to high concentrations of NO_3_^−^, Pb and As in certain products. Various regulations were rolled out in response to the ban (Yeo et al., 2021). The improved approaches for authentication and classification of EBN could benefit the whole industry by having a more economical, rapid, and streamlined industrial preparation of EBN products, as well as providing better guarantee of the quality of EBN products to meet the consumers requirements.

## Beneficial Health Functions of Edible Bird’s Nest

EBN has been a traditional, healthy food delicacy since the Tang Dynasty, however scientific research has been lacking over the last century. EBN-related publications remained scarce between 2000 and 2011, but the number of publications has increased significantly since 2012–2019. Discussions about the functions of EBN can be found in this review article by Lee et al. (2021) while our review will be focusing on recent discoveries of bioactive components in EBN and the functions of EBN that have never been discussed before.

### Antiviral, Immunomodulatory, and Antimicrobial Actions

EBN is known to exert antiviral bioactivity against influenza A virus (IAV) (Biddle and Belyavin, 1963). In another study carried out decades later, it was found that the non-pancreatin treated EBN extracts can bind to influenza viruses, but this antiviral effect was not seen in haemagglutination inhibition assay or the neutralization assay of influenza virus infected Madin-Darby Canine Kidney (MDCK) cells. The proteins or peptides which had the best anti-influenza effect were found to be sialylglycoproteins of size 10–25 kDa in the pancreatin treated EBN extract. Although proteins above 50 kDa (including a major allergen of 66 kDa) in the non-pancreatin treated EBN extract could bind to influenza A virus, neutralization against the virus was limited, indicating small molecular weight proteins of EBN extract was more favourable for its anti-viral effect. Furthermore, the EBN extract after pancreatin treatment did not trigger detrimental effects, such as hemolysis and cytolysis of erythrocytes and MDCK cells even at a higher concentration (4 mg/ml) (Guo et al., 2006). In summary, EBN extract treated with pancreatin is a more effective and safer antiviral agent when compared to non-pancreatin treated EBN extract.

Sialic acid (SA) and its derivatives found in EBN are highlighted by Guo *et al.*, in which they proposed that these compounds potentially contribute to the anti-influenza effects of EBN (Guo et al., 2006). Indeed, sialic acid is the most studied antiviral compound in EBN. One of the identified sialic acid is 5-N-acetylneuraminic acid (Neu5Ac) (Ogura, 2011). Interestingly, the Neu5Ac content in EBN is correlated with its potency in antiviral activity. The concentration of Neu5Ac varies among EBN samples collected at different geographic locations in Malaysia. EBN from Gua Madai contained 6.7 mg/g of Neu5Ac, which is higher than EBN collected from Teluk Intan which contained 3.2 mg/g of Neu5Ac (Haghani et al., 2017). SA residues could interact with the neuraminidase, and prevent this enzyme from cleaving the SA receptor present on the host cell surface hence halting the viral spread and release (Bianco et al., 2001; Haghani et al., 2016; Benton et al., 2017). Treatment with EBN for 24 h after the MDCK cells were inoculated with IAV for 1 h had the highest antiviral activity and percentage of protection against IAV when compared to EBN pre-treatment (MDCK cells treated with EBN for 1 h before IAV inoculation) and co-treatment (MDCK cells treated with EBN and IAV at the same time for 1 h) groups. It is speculated that the antiviral action of EBNs might be related to the viral release process from the cell membrane rather than viral attachment to the host cell. In the same study, EBN reduced early endosomal trafficking of the IAV by downregulation Rab5 and RhoA GTPase proteins. EBN also inhibited the IAV life cycle by regulation of autophagy with decreased expression of LC3-II and increased lysosomal degradation (Haghani et al., 2017). Besides that, EBN also reduced viral proliferation as effective as oseltamivir phosphate (a commercial antiviral drug) as shown by its effect in suppressing the viral enzyme, neuraminidase in IAV-infected BALB/c mice. Concomitantly, EBN showed high immunomodulatory effects against IAV by significantly increasing the levels of interferon-gamma (IFN-γ), tumour necrosis factor-alpha (TNFα), nuclear factor kappa B (NF-κB), interleukin-2 (IL-2), some pro-inflammatory cytokines (IL-1β and IL-6) and other regulatory cytokines (IL-4, IL-10, IL-12, IL-27 and CCL-2) depending on the stage of infection (Haghani et al., 2016).

At the same time, EBN also possesses immune-enhancing properties by modulating the immune system. However, there are only a limited number of experiments which have studied the effects of EBN on immune regulation. Zhao *et al.* studied the immunomodulatory effects of EBN in immunocompromised BALB/c mice treated by cyclophosphamide. Results showed that EBN stimulated proliferation and activation of B cells. Enhanced antibody secretion from B cells and better protection of the B cells from damage were detected in experimental mice, resulting in reduced intestinal immune damage (Zhao et al., 2016). Furthermore, EBN was also proven to enhance T lymphocytes transformation and increase serum immunoglobulin M (IgM) in the mouse model (Zhang et al., 1994). In addition, Cao *et al.* also stated that humoral immune, cellular immune and nonspecific immune were strengthened and regulated through EBN-induced improvements on the spleen and thymus index together with the phagocytosis rate and phagocytosis index of the peritoneal macrophages (Cao, 2012). Last but not least, EBN could also potentiate the proliferation of human peripheral blood monocytes (Ng et al., 1986).

EBN has also shown the potential in curing ulcerative colitis. An *in vivo* study was done by administering 5 ml 2% dextran sulfate sodium (DSS) via oral route daily for 7 days to induce ulcerative colitis in the male C57BL/6J mouse model. EBN was introduced in three concentrations (0.3 g/kg, 0.7 g/kg and 1.3 g/kg). Histologically, EBN-treated mice had a lower inflammation activity and a more complete structure of the submucosa, in comparison to the positive control group (without EBN supplementation) which showed excessive inflammatory cell infiltration, exfoliated epithelium, disrupted glandular arrangement and reduction in number of goblet cells in the submucosa. EBN reduced severe inflammatory reaction by lowering myeloperoxidase (MPO) activity and production of TNF-α and IL-6. In addition, reduced level of IL-17 and increased level of TGF-β were also determined in the EBN-treated groups. The author suggested that EBN ameliorated the inflammation by restoring the balance of Th17 cells and Treg cells, which was seen to be increased in the positive control group. This was further proved by the immunohistochemistry (IHC) and western blots, in which the IL-17A and Foxp3 genes were overexpressed in the positive control group whereas the overexpression of these two genes was inhibited by EBN. Concisely, EBN was proven to alleviate DSS-induced inflammation through immunomodulation by restoring the balance of Th17 and Treg cells and their associated cytokines (Fan et al., 2021).

Type of solvent used for extraction may have an impact on the antimicrobial activity of EBN. A metabolite, thymol-beta-D glucopyranoside was detected in the EBN. This metabolite has been previously reported to be an effective agent against food-borne bacteria (i.e. *Staphylococcus aureus (S. aureus), Escherichia coli (E. coli), Salmonella typhimurium, Shigella flexneri* etc.) (Liang et al., 2007; Chua et al., 2014). Hence, it was speculated that presence of this metabolite would confer antimicrobial activity in EBN. EBN extracted with methanol showed antibacterial activities for *S. aureus* (100 mg/L), *Candida albican (C. albican)* (100 mg/L), *E. coli* (1,000 mg/L), and *Aspergillus niger (A. niger)* (>3,000 mg/L), but the EBN extract derived by soaking extraction in ethyl acetate showed no antibacterial effects. Results of the solvent extraction methods showed that the EBN extracts with ethyl acetate were more effective than using methanol with *C. albican* (20 mg/L) and *A. niger* (20 mg/L) while the solvent extraction method with methanol showed slight effects with *S. aureus* (20–100 mg/L) and *E. coli* (>100 mg/L). In another study, the antibacterial activities of EBN extracted with 0.05 M alkaline with heating were tested on two Gram-positive (*Bacillus subtilis* and *S. aureus*) and two Gram-negative (*E. coli* and *Klebsiella pneumoniae*) bacterial strains but no antibacterial effects were observed in those tests (Saengkrajang et al., 2011). It is speculated that the extraction method could significantly affect the antibacterial effects of EBN. More studies should be done to identify the optimal extraction method of EBN to retain antimicrobial functions.

In short, EBN has shown its potential in enhancing the immune system in both *in vitro* and *in vivo* models. Nevertheless, more investigations are warranted to understand the mechanisms of immuno-stimulation and antibacterial effects exhibited by EBN.

### Cancer Prevention and Treatment

EGF were detected in the crude EBN collected from Rompin (30.7 pg/ml) and Sibu (74.5 pg/ml) from Malaysia using ELISA quantification kit. Tan et al. postulated that the presence of EGF in EBN can stimulate cancer cell growth. Four cancer cell lines (MCF-7 human breast adenocarcinoma cells, A549 human alveolar adenocarcinoma cells, Caco-2 human epithelial colorectal adenocarcinoma cells, and HCT116 human colorectal carcinoma cells) with EGF^+^ phenotype were selected to test for cell proliferative effects of EGF content in EBN using MTT assay. However, results showed no significant growth was observed in all 4 cell lines treated with EBN. It was thought that the EGF content in EBN could be too low to induce cancer growth (Tan et al., 2020). In another study, the cell viability of Caco-2 cell treated with two commercial EBNs were 84 and 115% respectively, while Caco-2 cells treated with unprocessed EBN from the East Coast, North and South Zones of Peninsular Malaysia were 91, 35 and 47% respectively. RAW 264.7 cell is a macrophage cell line that is usually used to study TNF-α expressions in macrophage. The EBNs collected from South and East Coast Zones of peninsular Malaysia as well as one of the commercial EBN, significantly reduced TNF-α production in RAW cells, where it was reduced to 24%, 32%, and 43% respectively. Geographical location and sources of the EBN are the factors resulting in the observed discrepancies in cancer cell proliferation rate. In the same study, commercial EBN products induced Caco-2 colorectal cancer cell growth. It is speculated that the adulterant in commercial EBN products stimulated Caco-2 cell growth, future studies are required to find out the substance responsible for promoting cancer cell growth in adulterants with introduction of better authentication procedure to detect such adulterants (Aswir and Wan Nazaimoon, 2011).

### Growth Factors and Tissue Regeneration

There is evidence that EBN possesses wound-healing effects. Hwang et al. studied the wound healing effects of EBN on HaCaTs cells and normal human dermal fibroblasts (NHDFs) which had been irradiated by ultraviolet B (UVB). Results showed that treatment with EBN significantly improved wound healing effects in both HaCaTs and NHDF cells. In fibroblasts, healing rate of the cells treated with 10 μg/ml increased by 39.6% after 24 h as compared to 0 h timepoint. Healing rate is faster in EBN treated cells as compared to fibroblastic self-healing (17% without any treatment) and 10 μg/ml allantoin treated group (28.2%). Hyaluronic acid, also known as hyaluronan, is a straight-chain carbohydrate extracellular matrix polymer that is a key component for the wound healing process (Aya and Stern, 2014). 10 μg/ml of EBN also significantly increased the production of hyaluronan in HaCaTs cells by 109.1%. In the study, EBN was also shown to confer anti-inflammatory effects as EBN reduced the expressions of two inflammatory cytokines, TARC/CCL17 by 89.7% and MDC/CCL22 by 46.1% in TNF-α/IFN-γ-stimulated HaCaTs cells. The author suggested that rapid healing effect induced by EBN was mediated through enhanced production of hyaluronan and the downregulation of MMP-1 and upregulation of procollagen type I expression in UVB-irradiated NHDF cells. In summary, these results indicate that EBNs have the potential to ameliorate UVB-induced skin photo ageing and TNF-α/IFN-γ-stimulated inflammation as well as wound injuries, resulting in faster healing rate (Hwang et al., 2020).

EBN also improves healing in the cornea. It was shown that rabbit corneal tissues treated with 0.05% EBN (with or without the addition of serum) resulted in increased expressions of collagen type I, aldehyde dehydrogenase (ALDH) and lumican, as compared to those treated with either serum-containing medium or serum-free medium (Zainal Abidin et al., 2011).

An *in vivo* study done by Albishtue et al. to study the effects of EBN on female reproductive system using adult female Sprague Dawley rats showed that oral EBN treatment at 60 and 120 mg/kg per day for 9 weeks augmented proliferation of uterine cells, including luminal epithelium, glandular epithelium, and stromal cells, resulting in better uterine and reproductive functions. Besides that, the endometrial receptivity and the number of implantation sites were also increased by EBN treatment. EBN also enhanced production of reproductive hormones, estrogen, progesterone, and prolactin, as well as expression of steroid receptors, progesterone and estrogen receptors. Upregulation of vascular endothelial growth factor (VEGF), EGF, EGF receptor, and proliferating cell nuclear antigen (PCNA) were found in the endometrial tissue. In short, EBN showed its potential to improve embryo implantation and also increased successful pregnancy rates (Albishtue et al., 2019).

Recently, it was reported that the action of Neu5Ac is not only limited to antiviral functions, but it also exhibits skin whitening and bone maintenance effects. In a skin whitening test, pepsin-digested EBN showed stronger inhibition of melanogenesis in cultured murine B16 skin cells and enzymatic activity of tyrosinase, as compared to that of undigested EBN. In addition, the pepsin-digested EBN also exhibited stronger osteogenic activity in cultured MG-63 osteoblasts cells. Neu5Ac in EBN was originally in conjugated form and was released from the conjugated form by treating EBN with pepsin in simulated gastric fluid at pH 2 condition for 48 h. This study indicated that prolonged EBN extraction in a gastric-like condition could achieve the full beneficial functions of EBN. Wong et al. tailored the method of Neu5Ac extraction from EBN (Wong et al., 2018c).

### Cardiometabolic Maintenance

EBN also has an impact on metabolism. Western blots showed that diabetic *db*/*db* mice treated with 75 and 150 mg/kg of EBN orally had elevated insulin levels when compared to mice treated with distilled water only. Meanwhile, insulin signalling receptor (IRβ) and downstream proteins (p-IRS1, PI3K and p-Akt) were also upregulated in mice treated with 75 and 150 mg/kg of EBN. Treatment with 75 and 150 mg/kg of raw EBN also decreased expression of pro-inflammatory cytokines, IL-6 and TNF-α, and also inflammatory protein, NF-κB in these mice. Moreover, at these doses, oxidative stress was ameliorated, as shown by downregulation of NADPH oxidase 4 (NOX4) protein, a reactive oxygen species (ROS) marker, and upregulation of superoxide dismutase-1 (SOD-1) protein, an antioxidant protein. In summary, EBN improves β-cell function and insulin signalling by attenuation of oxidative stress-mediated chronic inflammation in type 2 diabetic mice (Choy et al., 2021).

In another study, EBN demonstrated its potential role in preserving endothelial function by reducing oxidative stress in both cultured cells and mouse models. In the *ex vivo* study using male C57BL/6J mice, aortic ring assay indicated that impaired aortic relaxation in high glucose-fed mice was reversed with treatment of raw EBN (15 and 30 μg/ml). Human umbilical vein endothelial cells (HUVECs) were also utilized to examine the effects of EBN on high glucose-induced ROS formation. Results of lucigenin-enhanced chemiluminescence assay showed that treatment with 30 μg/ml of raw EBN significantly diminished intercellular ROS level and vascular superoxide anion production in HUVECs. EBN treatment at 30 μg/ml in HUVECs or 150 mg/kg in *db/db* mice reversed the high glucose-induced depletion of nitric oxide (NO). Western blot analysis also showed that EBN treatment at 30 μg/ml in HUVECs or 150 mg/kg in *db/db* mice could significantly reduce the level of NADPH oxidase 2 (NOX-2) and nitrotyrosine proteins while increase SOD-1 and p-eNOS protein levels (Murugan et al., 2020).

### Antioxidation and Neuroprotection

EBN has antioxidant effects which could serve as a novel alternative therapy for oxidative stress-mediated neurodegenerative diseases such as Alzheimer’s disease (AD) and Parkinson’s disease (PD) (Tai et al., 2017). The antioxidant capacity of EBN is higher in extracts obtained via alkaline extraction, it is speculated that the hydrolysed proteins release amino acids such as Cys, Met, His, Try and Lys with antioxidant properties (Lee et al., 2015). Simulated human gastro-intestinal digestion study demonstrated that the antioxidant components of EBN are released after digestion in the human gut. The digested EBN was shown to enhance antioxidative activities and protected HepG2 human liver cells from hydrogen peroxide (H_2_O_2_)-induced cytotoxicity (Yida et al., 2014).

Pathogenesis of PD involves oxidative stress-induced death of the midbrain dopaminergic neurons. EBN reversed ROS and nitric oxide (NO) build-up, reduced lipid peroxidation, inhibited caspase-3 cleavage, and attenuated apoptotic cell death in an *in vitro* PD model, neurotoxin 6-hydroxydopamine (6-OHDA)-treated SH-SY5Y cells. EBN improved motor function and balancing in 6-OHDA-treated C57BL/6J mice *in vivo* PD model. Inhibition of microglia activation and enhancement of antioxidant enzyme activity was shown in EBN-treated mice (Yew et al., 2014, 2018). Neurotrophic properties of EBN were demonstrated by increased cell proliferation and migration in a neural stem cell model, embryonic mouse neuroectodermal cells (NE-4C) (Yew et al., 2019). In another study, lipopolysaccharide (LPS) elicited cognitive impairment in rats by significantly increasing the escape latency while decreasing the number of entries in the probe trial, which were coupled with increased production of proinflammatory cytokines (TNF-α, IL-1β, and IL-6) and oxidative markers (ROS and TBARS) in the hippocampus. Treatment with EBN (125 mg/kg, 250 mg/kg, and 500 mg/kg; p.o.) effectively reversed effects of LPS on escape latency and probe trial. In addition, these treatments also inhibited the LPS-induced upregulation of pro-inflammatory cytokines and oxidative markers (Careena et al., 2018).

The effects of EBN on spatial learning and memory were also examined. The results showed that EBN supplementation had a dose-dependent improvement on cognition as evidenced by significant shorter escape latency. EBN-induced improvement of spatial learning and memory was also seen in the newly born offspring mice, which was fed on maternal milk of mother mice exposed to EBN supplementation. At the molecular level, EBN also exhibited its effect on attenuating neuro-inflammation and neuro-oxidative stress through increasing expression of SOD and decreasing levels of malondialdehyde (MDA) in the newly born offspring mice fed on maternal milk of mothers exposed to EBN supplementation. Evidence showed that EBN-induced enhancement of cognitive activity was due to elevation of SIRT1 expression in the pyramidal layer and dentate gyrus of the hippocampus. Besides that, EBN exhibited neuro-protective effect through anti-apoptotic mechanism in which the caspase-3 cleavage and early apoptotic membrane phosphatidylserine externalization were inhibited. The antioxidant effect of EBN through decreasing ROS level and increasing the expression of SOD gene in hippocampal neurons (SH-SY5Y neuroblastoma cells) was another neuroprotective mechanism (Ismail et al., 2021). In a recent study, zeroth generation CJ57BL/6 mice gave birth to first and second generations of offspring after 6 weeks of EBN supplementation (10 mg/kg). Both generations of offspring showed improvement in Y-maze cognitive performance at 6 weeks of age. Brain samples of the offspring mice demonstrated upregulation of GNE, ST8SiaIV, SLC17A5, and BDNF mRNA, and increased densities of synaptic vesicles in the presynaptic terminal (Mahaq et al., 2020).

Interestingly, EBN has also shown to have antioxidant effect in fly model. *Drosophila melanogaster* was cultured in four different groups, in which they were fed by food medium supplemented with 0 g/kg (control), 1 g/kg, 3 g/kg and 9 g/kg of EBN. Results of ferric reducing antioxidant power (FRAP) showed that the EBN-treated groups had higher total antioxidant activity in a dose-dependent manner as compared to control which had low total antioxidant activity. EBN enhanced the antioxidant capacity by increasing the SOD and catalase activities (CAT) whereas the MDA level was decreased. In a nutshell, EBN could improve ageing problems by reducing oxidative stress, hence increasing the lifespan of *Drosophila melanogaster* (Hu et al., 2016).

## Chemical and Biological Contaminants in Edible Bird’s Nest

Based on the Memorandum of understanding (MOU) on the Protocol of Inspection, Quarantine and Hygiene Requirements for importation of bird nest products from Indonesia and Malaysia into China, sealed and signed by Malaysia and Indonesia with China, the contaminants were assessed from the aspects of physical, microbiological, residual, heavy metals and excessive minerals, parameters and tolerance levels (Yeo et al., 2021). Bacteria, fungi, and mites are commonly reported to be found in EBN. Microbial growth in EBN might be attributed to the environment of EBN collection, such as high humidity levels and lower temperature or microbial infestation during EBN storage. The microorganisms could also be originated from saliva or feathers of swiftlets or the nest itself (Kew et al., 2014; Yeo et al., 2021). Additionally, Wong *et al.* revealed that both raw and commercial EBNs contain diverse types of bacteria, including *Staphylococcus sp.*, *Bacillus sp.* and *Acinetobacter sp.* Although the double-boiling treatment could effectively kill most of the bacteria, heat-resistant species like *Bacillus sp.* and *Brevibacillus sp.* were still isolated after treatment (Wong S. F. et al., 2018). Consumption of *Bacillus cereus* (*B. cereus*)-contaminated food can lead to diarrheal and emetic syndrome, resulting from production of *B. cereus* toxins (Griffiths and Schraft, 2017). A promising non-thermal processing method for food preservation by polychromatic low-energy X-ray with a high linear energy transfer (LET) effect results in a high relative biological effect (RBE). Low-energy X-ray with cut-off energy of 150 KeV was applied to inactivate two of the most prevalent foodborne pathogens in dry EBN. X-ray irradiation at 350 and 400 Gy decreased *E. coli* O157:H7 and *S. Typhimurium* from 6.35 ± 0.56 and 5.84 ± 0.67 log CFU/g, respectively, to undetectable levels. Based on dose distribution in 10 stacked pieces of EBN, two-sided irradiation could effectively inactivate pathogens uniformly (Zhang et al., 2020).

Environmental fungi from soil and plants are generally detected in both raw and commercial EBN. Although most of the fungi were removed after boiling of EBN up to 100˚C for 3 h, the environmental fungal genera of *Aspergillus sp.* and *Penicillum sp.*, can still be isolated in both EBN samples. It was speculated that these fungi were introduced from the EBN processing facility environment after the boiling process because these are two of the most frequently isolated environmental fungal genera. However, it is also possible that these fungal genera are thermoresistant or thermotolerant (Chen et al., 2015). *Aspergillus sp.* and *Penicillum sp.* are commonly known as food-spoilage fungi and can produce different mycotoxins (e.g. aflatoxins and ochratoxin A), which could cause various diseases in human as well as opportunistic infections in immunocompromised individuals (Greeff-Laubscher et al., 2020). Future studies are warranted for complete elimination and contamination management of these two fungal genera. Moreover, *Cladosporium* sp. and *Eurotium* sp. are detected in raw EBN samples, as these two genera of fungi are known to cause respiratory infections, swiftlet ranchers require personal protective equipment to prevent inhalation of these hazardous microbes (Chen et al., 2015). Mites are known as a source of allergens that could cause anaphylaxis (Sanchez-Borges et al., 1997). Mites, their faeces and eggs, and feather strands were observed on the surface of both raw and commercial EBN through EBN surface structural analysis under the electron microscope (SEM). These contaminants remained on the surface of EBN even after the washing and processing procedures were done (Kew et al., 2014; Tai et al., 2020). EBN is one of the causes of food-related anaphylaxis among children (Goh et al., 1999) and the pathophysiology is suggested to be related to immunoglobulin E (IgE)-mediated hypersensitivity caused by the protein existing in several isoforms seen at 66 kDa. N-terminal sequence of the major putative allergen (66 kD) showing homology to a domain of an ovoinhibitor precursor in chicken (Goh et al., 2001).

Insects consume plants inhabited by nitrogen-fixing bacteria and crops fertilized with nitrogen-based fertilizers. When swiftlets consume these insects, this results in accumulation of nitrites and nitrates in the swiftlets. As the nest is made of salivary secretion of swiftlets and embedded with droppings of swiftlets, high concentrations of NO_3_^−^ and nitrogen-fixing bacteria are detected. The nitrogen-fixing bacteria converts NO_3_^−^ into NO_2_^−^ giving rise to redding of EBN by NO_3_^−^ reductase. Although up to 98% of NO_2_^−^ could be removed after commercial EBN processing, NO_2_^−^ and NO_3_^−^ is still found in high concentrations in EBN, exceeding the acceptable tolerance level which is ≤30 ppm (But et al., 2013; Quek et al., 2018a; Yeo et al., 2021). In fact, the daily intake limit of NO_2_^−^ as advised by the World Health Organization (WHO) is between 0 and 3.7 mg/kg body weight (Yeo et al., 2021). To clear the traditional belief of red EBN being more precious, Paydar et al*.* (2013) discovered that the red colour in EBN is contributed by NO_3_^−^ and NO_2_^−^ instead of the presence of haemoglobin. NO_3_^−^ is relatively stable but NO_2_^−^ is active and can react with coexisting amino acids to form a carcinogenic compound called nitrosamine (Yeo et al., 2021). Semicarbazide, which originates from bleaching process used to remove impurities, was another carcinogenic compound found in EBN (Xing et al., 2012). A safer method to prevent red colouration of EBN is by using sodium tungstate, an inhibitor of NO_3_^−^ reductase that suppresses formation of NO_2_^−^ in cubilose (Chan et al., 2013). Nonetheless, a recent study reported that the acidic mammalian chitinase-like protein found in EBN contributes to the red colour as a noticeable increase in Fe-O bonding intensity after the colour change in EBN (Wong et al., 2018b).

Heavy metal and mineral contaminations in EBN were also reported in recent studies. Studies showed that in raw EBN, levels of Hg and Cu were higher than the permissible limits whereas the levels of Pb, As and Cd were below the limits (Chen et al., 2014; Quek et al., 2018b). A trace amount of these elements was also found in commercial EBN (Tan et al., 2020). As a matter of fact, the maximum permissible levels of each element is as follows; 1) Hg ≤0.05 ppm; 2) Cu ≤1 ppm; 3) Pb ≤2 ppm; 4) As ≤1 ppm; and 5) Cd ≤1 ppm. In addition Fe level was found to be higher than the regulatory limit (0.3 ppm) in both raw and commercial EBN (Yeo et al., 2021). All these heavy metals, if consumed in excessive amounts could react with proteins or enzymes in the human body, causing chronic heavy metal poisoning syndrome (Dai et al., 2020). For example, Hg intoxication could cause severe behavioural and cognitive changes as well as delayed development of growth and neural system in children (Ha et al., 2017). Other than that, Cd is known to be a mutagenic compound and Cd consumption could result in cancer development (Fatima et al., 2019). An excess amount of Cu is also associated with certain human disorders, such as cardiovascular diseases, neurotoxicity and hepatic disease (But et al., 2013; Paydar et al., 2013).

In short, EBN consumption undoubtedly has its safety issues due to the potential residual contaminations including microorganisms (bacteria, fungi, and mites), heavy metals (Hg, Pb, As and Cd), minerals (Fe and Cu) as well as NO_2_^−^ and NO_3_^−^ contents. On top of that, adulterants used in EBN production and semicarbazide used in the bleaching process could also pose hazardous effects to human health. Therefore, the quality and authentication of EBN should be strictly regulated to avoid consumers from eating inauthentic EBN and thus the risk of EBN consumption could be reduced to a greaer extent.

## Edible Bird’s Nest Processing

The study of EBN has recently gained attention from researchers in the past decade as recent laboratory studies of EBN demonstrated many optimisation steps for the production of better EBN products. We summarise the optimisation steps based on laboratory findings that could be useful references for setting up the industrial EBN processing with the aims to improve the safety, taste, and preserve the health benefits of EBN products (Table 1).

**TABLE 1:** Summary of EBN extraction studies.

Authors	Extraction methods	Nutrients	Bioactive components	Location	Functions
Yew et al. (2014)	Water extraction	-	-	Malaysia: Perak swiftlet premises	Increase antioxidant properties, Inhibit early apoptotic membrane phosphatidylserine externalization, Inhibition of caspase-3 cleavage
	Pancreatin enzymatic extraction	-	-	-	Improves cell viability of 6-OHDA-challenged SH-SY5Y cell
Yew et al. (2018)	Water extraction and Pancreatin enzymatic extraction	-	-	Malaysia: Perak swiftlet premises	Improves motor function and balancing in PD mice, Prevents 6-OHDA-induced loss of dopaminergic neuron in substantia nigra of PD mice, Improves antioxidant in PD mice, Reduces microglia activation in PD mice, Reduces NO production and lipid peroxidation in PD cell
Yew et al. (2019)	Water extraction and Pancreatin enzymatic extraction	-	-	Malaysia: Perak swiftlet premises	Promoting proliferation and migration of the NSC model, embryonic mouse neuroectodermal cells (NE-4C)
	Water extraction	-	Repulsive guidance molecule domain family member B, Protein lin-9, and hyaluronan mediated motility receptor	-	Promote neurite extension, axonal growth, cell proliferation and migration
Tong et al. (2020)	eHMG	193 metabolites	-	Malaysia: Johor swiftlet premises	-
	pHMG	42 metabolites	-	-	-
	Sulfuric acid extraction	6 metabolites	-	-	-
	Pancreatin enzymatic extraction	1 metabolite	-	-	-
Zainal Abidin et al. (2011)	eHMG	-	Collagen Type I		Major structural collagen of the cornea
			aldehyde dehydrogenase (ALDH)		Oxidation of a wide variety of endogenous and exogenous aldehydes to their corresponding acids
			Lumican		Normal cornea morphogenesis
Khushairay et al. (2014)	Heat and Pancreatin enzymatic extraction	Protein solubility to 163.9 mg/g, Degree Hydrolysis: 86.5% and 109.5 mg/g peptides	-	-	-
	Heat and Alcalase enzymatic extraction	Protein solubility of 86.7 mg/g, Degree Hydrolysis 82.7% and 104.1 mg/g peptides	-	-	-
Ling et al. (2020)	Protease from *Bacillus licheniformis*	Soluble protein: 375.1 ± 4.7 g/kg	Sialic acids: 126.1 ± 4.0 g/kg		Increase antioxidant activity
Guo et al. (2006)	Neuraminidase from Clostridium perfringens	-	*N*-acetylneuraminic acid	Indonesia: cave and swiftlet premises	Neutralize the infection of MDCK cells with influenza viruses, Inhibit hemagglutination of influenza viruses to erythrocytes
Tai et al. (2020)	Bromelain extraction		Bromelain from pineapple		Removal of mites
Amiza et al., 2019a	Heat extraction	Protein Hydrolysis: 5.84–14.54%	-	-	-
	Heat and Alcalase enzymatic extraction	Protein Hydrolysis: 12.16–22.59%	-	-	-
Hun et al. (2020)	Modified water extraction with ethyl acetate, n-butanol, and acetone-water mixture	54 metabolites	Triglycerides	Malaysia: Johor	Improve antioxidant antivities
Fan et al. (2020)	Dynamic high pressure microfluidization (DHPM)	Protein: 69.56 ± 1.74%, Carbohydrate: 17.56 ± 0.45%, Sialic acid: 10.77 ± 0.42%	-	-	-
Wong et al. (2018c)	Pepsin and simulated gastric fluid at pH 2	-	N-acetylneuraminic acid	Indonesia: swiflet premises, Malaysia: swiftlet premises, Thailand: cave, Vietnam: cave	Inhibition of melanogenesis, Stronger enzymatic activity of tyrosinase, Stronger osteogenic activity
Haghani et al. (2016)	Heat extraction, heat and enzymatic extraction	-	Sialic acids and thymol derivatives	Teluk Intan, Perak, Malaysia: swiftlet premises, Gua Madai, Lahad Datu, Malaysia: cave	Heat extraction stronger effects than heat + pancreatin enzymatic extraction: Decrease NS1 and NA gene copies of IAV
Haghani et al. (2017)	Heat extraction, heat and enzymatic extraction	-	a2,3-N-acetylneuraminic acids (sialic acids): Gua Madai = 6.7 mg/g, Teluk Intan = 3.2 mg/g	Teluk Intan, Perak, Malaysia: swiftlet premises, Gua Madai, Lahad Datu, Malaysia: cave	Reduce Rab5 activity, Inhibit autophagy, Increase lysosomal degradation
Fan et al. (2021)	Heat extraction	-	-	-	Decrease expression of IL-1β, TNF-α, IL-17A, and IL-6, Increase expression of TGF-β, Restore Th17/Treg cell balance in intestine of C57BL/6J Mice
Saengkrajang et al. (2011)	Methanol extraction	-	-	-	Inhibits growth of *S. aureus, C. albican, E. coli, A. niger*
	Ethyl acetate extraction	-	-	-	Inhibits growth of *C. albican and A. niger*
Tan et al. (2020)	Heat, pepsin, and pancreatin extraction	protein: 53.03–56.37% and carbohydrate: 27.97–31.68%	EGF	Malaysia: Alor Setar, Kedah. Sibu, Sarawak. Rompin, Pahang. Kuala Selangor. Johor Bahru. Jerantut, Pahang. Port Klang	Insignificant changes of cell viability in MCF-7, A549, Caco-2, HCT116 cells
Aswir and Wan Nazaimoon, (2011)	Heat and hydrochloric acid extraction	-	Sialic acids	North, South, and East coast of Peninsular Malaysia	Increase Caco-2 cell proliferation and reduce TNF-α production
Hwang et al. (2020)	Water extraction	-	-	A: Java city, B: Sumatra/Banka cities	Increase antioxidant properties, Decrease TNF-α, IFN-γ, and matrix metalloproteinase-1 expression, Increase expression of hyaluronan, Increase procollagen type I synthesis
Albishtue et al. (2019)	Heat extraction	-	-	-	Improve embryo implantation and pregnancy rates in Sprague Dawley rats, Increase productions of hormones and hormone receptors, Increase antioxidant properties
Choy et al. (2021)	Heat extraction	17 metabolites	Sialic acids	-	Increase expressions of insulin and insulin receptors, Decrease inflammatory cytokines expression, Increase antioxidant properties in *db/db* diabetic mice
Murugan et al. (2020)	Heat extraction	-	Sialic acids: 1.26 μg/mg	-	Preserve endothelial functions in glucose-treated C57BL/6J mice, Increase antioxidant properties in *db/db* diabetic mice
Lee et al. (2015)	Heat and alkaline extraction	Proteins:35.8% ± 0.12	-	Batu Pahat, Johor, Malaysia	Increase antioxidant properties and no antibacterial function
	Sodium Chloride extraction				
	Hydrochloric extraction				
Yida et al. (2014)	Heat extraction				
	Heat, pepsin, pancreatin, and bile extracts extraction (pH 8.9 -> 2 -> 8.9)	-	-	-	Protect HepG2 cells from hydrogen peroxide-induced toxicity by increasing antioxidant activity
Careena et al. (2018)	Heat, pepsin, and pancreatin extraction at pH 2	-	Sialic acids	Malaysia: East, southern, northern, western coast of Peninsular Malaysia, heavily polluted industrial area, and East Malaysia	Improve brain functions by Increasing antioxidant properties and decreasing proinflammatory cytokines in hippocampus in LPS-induced Wistar rats
Mahaq et al. (2020)	Heat extraction	-	Sialic acids	Malaysia: North and South Peninsular Malaysia, Sabah	Improve spatial recognition memory in next two generations offsprings of CJ57BL/6 mice: Upregulation of GNE, 4ST8SiaIV, SLC17A5, and BDNF mRNA. Increase densities of synaptic vesicles in the presynaptic terminal
Hu et al. (2016)	Water extraction	-	Sialic acids: N-acetyl neuraminic acid 5.4%	Vietnam: Nha Trang	Increase lifespan, Decrease mortality rate and increase survival rate against heat-stress test, Increase antioxidant properties
Quek et al., 2018	Heat extraction	-	Sialic acids: 13.6 g/100 g	Peninsular Malaysia: Segamat, Johor. Kapar, Selangor. Nibong Tebal, Penang. Klang, Selangor	Increase antioxidant properties
			Sialic acids: 9.1 g/100 g	East Malaysia: Sarikei, Sarawak. Gomantong Cave, Sabah. Baram, Sarawak. Niah Cave, Sarawak. Subis Cave, Sarawak	
Norhayati et al. (2010)	Hydrochloric acid extraction	Proteins: 61.5 ± 0.6 g/100 g, Calcium: 553.1 ± 19.5 mg/100 g, Sodium: 187.9 ± 10.4 mg/100 g, Magnesium: 92.9 ± 2.0 mg/100 g, Potassium: 6.3 ± 0.4 mg/100 g	Sialic acids: 0.7–1.5%	-	-
Halimi et al. (2014)	Heat and hydrochloric acid extraction	Proteins: 58.55%, Carbohydrate: 22.28%, Fat: 0.67%	-	Pahang, Malaysia	-
		Proteins: 55.48%, Carbohydrate: 25.79%, Fat: 0.29%	-	Terengganu, Malaysia	-
Chua et al. (2014)	Chloroform + methanol (1:1) extraction with sonication	43 metabolites	-	Malaysia, Indonesia, and Thailand	-
	Methanol extraction with sonication	35 metabolites			
Saengkrajang et al. (2013)	Hydrochloric acid and chloroform + methanol (1:1) extraction	Proteins: 61.0–66.9%, Essential amino acids: 15.9–31.6 mg/g, Carbohydrates: 25.4–31.4%	-	Thailand: Trat province, Phetchaburi province, Nakhon Si Thammarat, Satun, and Narathiwat provinces	-
Elfita et al. (2020)	Heat and hydrochloric acid extraction	Proteins: 53.09–56.25%, Carbohydrate: 19.57–23.04%, Fat: 0.07–0.57%	-	Indonesia: West Sumatra, South Sumatra, West Java, West Kalimantan, Central Sulawesi, and Southeast Sulawesi	-
Phyu Win et al. (2020)	Heat extraction	Proteins: 53.5–59.3 %	-	Myanmar	-

Table legend: “-” indicates “not studied”. Glucosamine (UDP-N-acetyl)-2-epimerase/N-acetylmannosamine kinase (GNE), ST8 alpha-N-acetyl-neuraminide alpha-2,8-sialyltransferase (ST8SiaIV), solute carrier family 17 member 5 (SLC17A5), and brain derived neurotrophic factor (BDNF).

### Laboratory and Traditional Industrial Edible Bird’s Nest Processing

After collection from the swiftlet premises or caves, EBN is processed following these general procedures: cleaning, drying, grinding, soaking/elution (specific for elution water extraction (eHMG)), heating, acid/alkaline treatment (specific for acid/alkaline extraction), enzymatic treatment (specific for enzymatic extraction), filter, centrifugation, freeze-drying/lyophilisation, storage. Slight changes for processing steps were made as reported in different groups for the consideration of targeted nutrients, convenience, cost-effectiveness, and instruments availability. These processing steps are critical for preservation of nutritional values of EBN (Yew et al., 2014; Gan et al., 2017b; Hong et al., 2020; Tong et al., 2020).

Firstly, the collected EBN is soaked in ultrapure water until softened to loosen the protein strands and cleaned by washing in water or ultrapure water, followed by manual removal of dirt, feathers, and egg shells using forceps (Yew et al., 2014). The dust and lighter impurities floating in the water and EBN could be easily picked with forceps as the EBN expands in size after soaking in water (Hong et al., 2020). Then, the cleaned EBN is dried with or without controlled air circulation, heat, and humidity. EBN could be dried at a faster rate with higher air circulation speed and temperature but this intervention is associated with reduced sialic acid and antioxidant retention in EBN (Gan et al., 2017a; 2017b). The dried EBN, around 10–12% moisture then undergoes grounding with pestle and mortar manually or electric blending based on preferences as no study was conducted for the comparison between manual and automatic grounding process (Gan et al., 2017a; Yew et al., 2019). The grounded EBN is sieved through a wire mesh (0.4–1.0 mm) to further separate the feathers and other impurities (Zainal Abidin et al., 2011). The next step is extraction of chemical compositions of EBN by extraction method of HMG, heat extraction, enzymatic extraction, acid extraction, alkaline extraction, and eHMG.

Grounded EBN is soaked in cold distilled water 2.5% (w/v) or deionized water at 0.2% (w/v) at 4˚C, and usually left for overnight but some studies have also left it for 16–48 h (Guo et al., 2006; Yew et al., 2014; Tong et al., 2020). After that, heating of EBN solution is carried out in the range of 40˚C to 121˚C for 15 min to 4 h (Amiza et al., 2019a; Tong et al., 2020). If it was acidic extraction, the EBN mixture is heated at 80°C with 2% (v/v) of 0.4 M sulfuric acid for 4 h subsequently allowed to cool down and centrifuged at 2,716 g (5,000 rpm) for 15 min. The pH of supernatant collected is neutralized to pH 7.0. White precipitation formed is removed through centrifugation with 2,716 g (5,000 rpm) for 15 min at 4°C. The supernatant is then collected and kept at 4°C for further analysis (Tong et al., 2020). However, if it was an alkaline extraction, 1 g of each raw sample is immersed in 30 ml of 0.1–0.4 M NaOH solution for 48 h. Then, the aliquot of each extract is immersed in the water bath at 65°C for 2 h. The extracted solutions are centrifuged and eventually the supernatant is obtained. After the extraction process, the suspensions are centrifuged at 18,000 rpm for 20 min, and the supernatants are thoroughly dialysed against distilled water (Hun et al., 2016). For enzymatic treatment, enzymes such as pancreatin, alcalase (Khushairay et al., 2014), protease from *Bacillus licheniformis* (Ling et al., 2020), and neuraminidase from *C. perfringens* (Guo et al., 2006) is used. In continuation of the heating, the enzyme is usually added to the EBN mixture at a warm temperature between 38°C-60°C, at pH 8.5–9.0 to facilitate partial protein denaturation. It is then followed by heating at 90°C for 5 min for enzyme deactivation. The EBN mixture is then filtered using filter paper, filtrate is collected and freeze-dried to obtain EBN powder (Guo et al., 2006; Yew et al., 2014). Freeze-drying process aims to produce dehydrated EBN powder while retaining maximum nutrient and bioactive compounds (Bhatta et al., 2020). Lastly, the freeze-dried EBN powder is stored at 4°C or −80°C freezers for future use. In industrial setting, freeze-dried EBN could be made into cosmetics, tablets, ready-to-drink beverages, tonics, spices and baking powder among others (Lee et al., 2021). In the industrial processing of whole raw EBN, it is started with sorting and grading of EBN based on the difficulty to clean the EBN. The cleaning step is important as EBN with more feathers and impurities are graded lower and sold at a lower price. After sorting, EBN will be soaked in clean water, so that dust and impurities will float on the water. Feathers that remained on the EBN will be hand-picked with forceps or tweezers. This is a labour-intensive process that could be time-consuming. After that, EBN is placed on a mold and left to dry by heating cabinet and fan blowing. This process could lead to nutrients loss. The dried EBNs in cup shape is sorted and graded again based on their colour, cleanliness, and shape for packing (Hong et al., 2020).

### Industrial Edible Bird’s Nest Processing Optimised With Laboratory Findings

As mentioned earlier, EBN is required to be free from avian influenza. The MOU on the Protocol of Inspection, Quarantine and Hygiene Requirements demands EBN products from Indonesia and Malaysia to be free from avian influenza to be imported into China (Badan Karantina Indonesia, 2014; Rohaizan, 2017). Only EBN obtained from farms registered with the Department of Veterinary Services, Malaysia is authenticated. The Chinese government has imposed stringent procedures for quality assurance by tracing the entire supply chain from EBN harvesting to exporting to China (Thorburn, 2015). Additionally, EBN processing facilities must be inspected and approved by Certification and Accreditation Administration of the People’s Republic of China (CNCA) (Minister Department, 2012). According to the information provided by the CAIQ official website http://ebn.caiq.org.cn/overseasRegist, Indonesia has 23 EBN processing facilities and 436 swiftlet houses approved to be exported to China; Malaysia has 41 EBN processing facilities, but there is no information about the registered swiftlet houses; Thailand has 2 EBN processing facilities and 21 registered cave EBN sources (Development Report of Imported Bird’s Nest in 2021, 2021).

Department of Veterinary Services of the Ministry of Agriculture and Food Safety and Quality Division, Ministry of Health, Malaysia, implements and enforces that EBN products must be processed with heat treatment, with core temperature of the products shall be higher than 70°C and retained for at least 3.5 s to effectively kill the avian influenza virus (Yeo et al., 2021). Apart from that, environmental fungi in raw EBN should be removed before consumption. Heating EBN to ≥100°C for at least 3 h could remove the environmental fungi in EBN (Chen et al., 2015). The heating temperature and time possess critical effects on extraction of bioactive compounds from EBN as well as retention its nutritional values. Excessive heat treatment is known to cause alteration in protein native structure resulting in unfolding of protein and altered protein surface exposure (Teodorowicz et al., 2017). As the proteins denature, bioactive properties are lost and this would affect its nutritional value.

Furthermore, differential scanning calorimetry have often detected loosely bound water (dehydrates from EBN below 110˚C) and tightly bound water (dehydrates from EBN between 100˚C and 200˚C) in EBN samples. In fact, it implies the wide use of H_2_O_2_, as a bactericide and bleaching agent in bird nest cleaning industry to make the EBN more whitish, so that it could be graded as high-grade EBN. Some of the H_2_O_2_ may be tightly hydrogen-bond in coils of the glycoprotein chains, just like the tightly bound water, and may remain trapped in the EBN on drying typically below 60°C, and this could present as a food hazard. It may be necessary to heat dry the cleaned EBN at above 100°C to dislodge and decompose H_2_O_2_, or to reduce it in the moist EBN with permitted reducing agents like ascorbic acid, or by enzymatic decomposition with catalase, before drying (Shim et al., 2017). This study further supports the importance of heat treatment for EBN. Future studies focusing on the optimum heating condition for pathogenic microorganisms removal and nutrients retention should be carried out. The heating step should be introduced into industrial EBN products processing, especially for those ready-to-drink beverages to prevent potential avian influenza virus infection and food poisoning.

Mites and fungal spores are contaminants widely found in EBN and more mite faecal pellets and eggshells were found in raw EBN compared to commercially processed EBN (Kew et al., 2014). Certain mites are heat-resistant and can survive the cooking process to elicit allergic reactions in consumers (Yan et al., 2008). Bromelain, an enzyme derived from pineapple showed promising results in killing mites and fungi found in raw EBN (Tai et al., 2020). Using bromelain to remove microbial contaminants could be a safer choice than bleaching agents like H_2_O_2_ and semicarbazide. A study showed that semicarbazide detected in commercial EBN products originated from bleaching of EBN. As semicarbazide is carcinogenic, use of semicarbazide to bleach EBN should be strictly prohibited (Xing et al., 2012). Moreover, the packaging of EBN products should avoid using azodicarbonamide-containing packaging as it could produce semicarbazide due to thermal decomposition (Stadler et al., 2004). Most importantly, adulterants should not be added to EBN products and storage should be kept clean from potential contaminants for safe consumption of EBN. Hypothetically, simultaneous applications of both heat and enzymatic EBN extraction could provide additional advantages after optimisation.

Apart from using heat and enzymatic treatments to remove contaminants, these steps could improve bioavailability of nutrients in EBN. The extracted product compositions are unique for each extraction method due to their physicochemical properties (Tong et al., 2020). Protein functionalities based on its physical and chemical properties including size, shape, amino acid composition, sequence, net charge and distribution, hydrophobicity/hydrophilic ratio, secondary, tertiary, and quaternary structures, molecular flexibility/rigidity, and ability to interact with other components. However, extensive denaturation of proteins often results in insolubilisation, which can consequently impair functional properties that are dependent on solubility. Ideally, partial denaturation of protein often improves digestibility and biological availability of essential amino acids (Damodaran and Parkin, 2017). Enzymatic protein hydrolysis is commonly used to modify the nutritional, physicochemical, functional, digestibility, sensory, and bioactive properties of the protein, as well as to reduce allergenic and anti-nutritional compounds (Tavano, 2013).

A widely studied enzyme, pancreatin is a mixture of digestive enzymes that is secreted from the pancreas with proteolytic, amylolytic and lipolytic activities. Treatment with pancreatin significantly increases the protein’s solubility, degree of hydrolysis and bioactive peptides concentration of the mixture (Khushairay et al., 2014). For example, pancreatin-treated EBN has higher efficiency compared to non-pancreatin treated EBN in reducing cell death in 6-OHDA-challenged SH-SY5Y cells. However, water extract exhibited higher efficacy in ameliorating ROS build up, early apoptotic membrane phosphatidylserine externalization as well as inhibition of caspase-3 cleavage (Yew et al., 2014). In *in vitro assay,* EBN treated with pancreatin had superior anti-viral effect and showed no side effects, rendering the pancreatin treated EBN a safer and more effective antiviral agent as compared to EBN extracted with water (Guo et al., 2006).

The choice of EBN extraction should be made based on desired biomedical application because bioactive compounds could be best extracted by a particular extraction method. Alcalase is an enzyme that has broad specificity and has been employed to modify the functional properties of a range of proteins. It is used mainly to cleave the carboxyl side of the hydrophobic amino acid (Adler-Nissen and Olsen, 1979). It is speculated that combined applications of heat treatment and enzymatic protein hydrolysis may help to maximise bioactive components functionality, digestibility, and bioactivity. When compared to a variety of heating modalities and heating plus alcalase treatment: slow cook with or without alcalase treatment (85–96˚C, 60–120 min), autoclave with or without alcalase (121˚C, 15 min), and raw (without heating and alcalase treatment), EBN heated at 100˚C for 60 and 30 min followed by alcalase treatment gave highest degree of hydrolysis, 22.59 and 20.42% respectively. The degree of hydrolysis is positively correlated with solubility where high solubility of normal boiled, enzymatically hydrolysed EBN possess the potential of EBN being used for food formulation, nutraceutical, and cosmetic products (Amiza et al., 2019a). On top of that, alcalase treatment also helped in removal of impurities (for example ash content), and improves the aesthetics of commercially available EBN (Noor et al., 2018).

Neu5Ac in EBN is known for its antiviral, skin whitening, and bone maintenance functions. The EBN treated with pepsin in simulated gastric fluid at pH 2 for 48 h showed higher potency for the functions as mentioned than those EBN samples without extraction process under such condition. Hence, prolonged treatment of EBN in gastric-like conditions could retain better Neu5Ac bioactivity (Wong et al., 2018c). The application of enzymes in EBN preparation have shown promising results and it is anticipated that these procedures should be incorporated into industrial EBN processing. Treating EBN with enzymes for elimination of contaminants, extraction of bioactive compounds, and allergen deactivation could provide consumers with better and safer EBN products.

The pH value during EBN extraction is one of the important factors to retain maximum bioactivity. Both acid and alkaline extraction methods including hydrochloric acid (Quek et al., 2018a) and sodium hydroxide (Hun et al., 2016) were studied. Extracting bioactive compounds from EBN using acid was shown to be inefficient when compared to acidic enzymatic extraction. Hence, supplementing enzymatic extraction (e.g. pepsin) with addition of acids facilitates protein denaturation and the EBN extract will be neutralized to achieve a pH of 7.0 with alkaline to halt the enzyme activity (Tong et al., 2020). In contrast, Amiza et al. (2019b) demonstrated that the optimum conditions for EBN hydrolysis is at pH of 9.46, Alcalase to substrate concentration of 2%, hydrolysis time of 179.55 min, and 64.99°C, can achieve 37.9% degree of hydrolysis. Alcalase require pH 7.5–9.5 and neutralized with acids after the extraction (Amiza et al., 2019b).

The fatty acids content of EBN is relatively understudied but recently Lee et al. reported that EBN contains 48.43% of poly-unsaturated fatty acids (PUFA), 25.35% of saturated fatty acids (SFA), and 24.74% mono-unsaturated fats (MUFA). These fatty acids explain the antioxidative activity of EBN. Briefly, after HMG was done, the triglyceride fraction from EBN was extracted with acetone water mixture (ratio of 75:25) at room temperature for 2 days. The aqueous solution was then obtained after solvent evaporation under a vacuum that was successively partitioned with ethyl acetate (Hun et al., 2020). One of the important factors for EBN nutrients retention is the pH value during acid extraction (Hun et al., 2020).

Recent studies have reported new extraction methods with high efficiency in extracting the highest amount of nutrients from EBN. The EBN eHMG extraction was first demonstrated by Oda et al. and remained a non-disclosure procedure to extract EBN (Oda et al., 1998). Recently, eHMG was found to be the more effective than acid extraction, enzyme extraction, and HMG in terms of EBN extraction, with the highest number of water-soluble (193) metabolites and extra sialic acids extracted (Tong et al., 2020). A recent study showed that the EBN processed with dynamic high-pressure micro-fluidisation (DHPM) at various pressure (range from 20–200 MPa) displayed a remarkable increase in protein solubility. DHPM treatment significantly improved the solubility accompanied by changes in the structural properties of EBN insoluble proteins, which may be caused by intense shear, turbulence force, and cavitation effect of DHPM treatment. Confirmational changes such as the increase in α-helix and β-turn, reduced particle size and de-aggregation of large proteins in EBN water-insoluble fraction. Hence, optimized DHPM could be an alternative method to traditional stew to release the bioactive compound from EBN (Fan et al., 2020). Mass eHMG and DHPM EBN processing could be developed and tested in the future.

**FIGURE 1:**
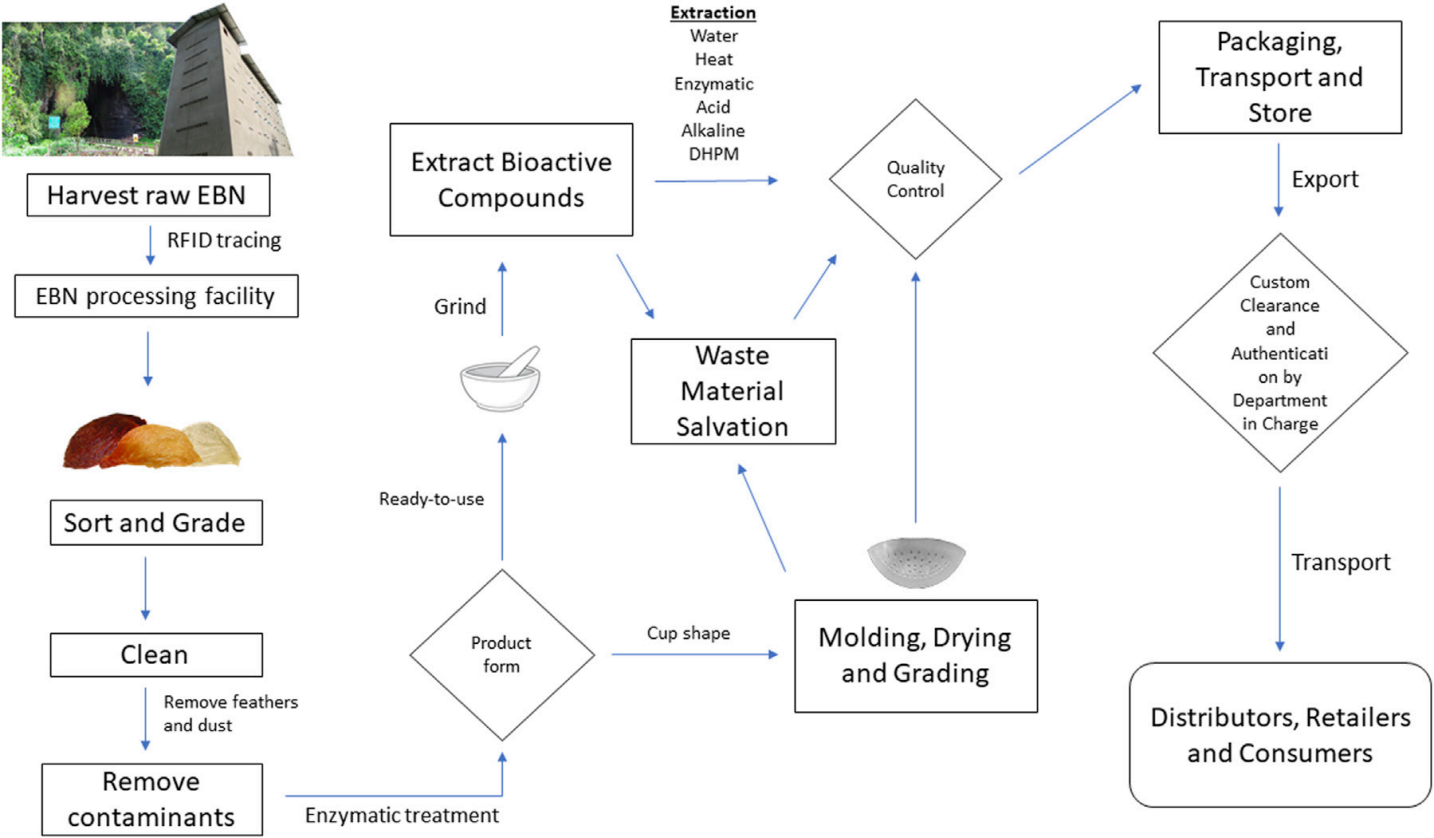
Schematic flowchart of EBN industry.

Based on the common practice of current whole EBN processing, whole EBN is dried on a mold after soaking and cleaning, skipping extraction as shown in Figure 1 (Hong et al., 2020). The drying process after cleaning of EBN was discovered to be critical for sialic acids and antioxidants retention, in turn providing benefit to the quality and grade of EBN. Higher drying temperature causes degradation of sialic acids and antioxidants in EBN, exhibiting first-order kinetics. EBN dried at 25°C had 83.9 and 96.6% retention of sialic acids and antioxidants, 78.7 and 91.5% at 40°C, 42.5 and 38.7% at 70°C respectively. Longer drying time is required for drying at lower temperature to achieve similar level of dryness in the EBN as the processing of EBN would be slowed down but the retention of nutritional value is significantly better. When compared to convective hot air drying, heat pump drying at a temperature lower than 40°C, with forced air in the initial stage of drying and intermittent forced air supply in the later stage could efficiently retain colour and sialic acid in EBN. Traditional EBN drying step could be improved as suggested by Gan et al. to preserve the highest possible amount of nutrients in EBN. Temperature as low as 25°C could be used to best preserve the colour, antioxidants, and sialic acids in EBN although it will take a longer time to dry and therefore affects the turnaround time for the industrial preparation process. In summary, heat pump drying at a temperature lower than 40°C, with forced-air in the initial stage of drying and intermittent forced air supply in the later stage could efficiently retain colour, antioxidants, and sialic acids in EBN (Gan et al., 2017a; 2017b).

Surprisingly, around 50% of glycopeptide can be recovered from the waste material of EBN, particularly N-acetylneuraminic acid, which is 229% higher than EBN hydrolysate after enzymatic extraction. The antioxidant activities found in waste material after enzymatic extraction was higher than the EBN extract, suggesting a significant bioactive portion of EBN was wasted during the extraction process. The cleaning procedure is a time-consuming process that includes immersion, swelling, separation of impurities, sterilization, molding, drying and secondary sterilization. The cost of processed clean EBN was approximately 2200–2500 USD kg^−1^ and unprocessed EBN was 1200–1500 USD kg^−1^ in 2017, and this value is expected to increase in the future. The salvation step should be included in enzymatic extraction procedure to prevent economical and nutritional value loss (Ling et al., 2020).

## Conclusion

This review compiled recent investigations of EBN and provided insight into various topics related to EBN products. Increasing studies have proven beneficial health effects of EBN consumption but factors, such as geographical location, harvesting place, harvesting season, and processing influences the nutritional contents of EBN. Hence, standardised EBN processing methods are required to preserve the bioactive effects discussed in this review. The health concerns of EBN consumption caused by adulterants, chemical and microbial contamination should be addressed by strict adherence to guidelines. Several studies demonstrated plausible authentication methods could be translated into industrial settings, but cost-effectiveness and equipment availability required further analysis and comparison. Numerous *in vivo* animal studies showed promising health-improving functions of EBN with discoveries of bioactive compounds. Future studies to fill the knowledge gap of EBN in promoting human health is promising.
